# Harnessing CRISPR-Cas9 for *Lactobacillus* improvement in silage production: current knowledge and future perspectives

**DOI:** 10.1186/s40104-025-01282-x

**Published:** 2025-11-15

**Authors:** Jing Ma, Jiao Zhang, Xusheng Guo

**Affiliations:** 1 School of Life Sciences, Lanzhou University, Lanzhou, Gansu, 730030 P.R. China; 2 Probiotics and Life Health Institute, Lanzhou University, Lanzhou, Gansu, 730030 P.R. China; 3 School of Life Sciences and Engineering, Northwest Minzu University, Lanzhou, Gansu, 730030 P.R. China

**Keywords:** Gene editing, *Lactobacillus* engineering, Silage, Sustainable livestock production

## Abstract

High-quality silage is the cornerstone to sustainable livestock development and animal food production. As the core fermentation bacteria of silage, *Lactobacillus* directly regulates silage fermentation by producing lactic acid, enzymes, and other bioactive molecules. However, traditional screening methods for functional strains are labor-intensive and time-consuming. Recent advances in synthetic biology, particularly the development of CRISPR-Cas genome editing technology, offer a revolutionary approach to designing *Lactobacillus* strains with customized traits. This review systematically reviewed the importance of silage in sustainable agricultural development and the limitations of current silage preparation and promotion. It also discussed the application of strain engineering approaches in optimizing the phenotypic performance of *Lactobacillus* for better silage. Building on this, we reviewed the research progress of CRISPR-Cas9 gene editing in *Lactobacillus* and discussed how to leverage its high efficiency and precision to optimize the strain’s traits for improved silage quality and functionality. CRISPR-Cas9 toolkits are expected to achieve directed evolution of strain performance, ultimately yielding next-generation silage microbial inoculants with multiple functions, adaptability to multiple substrates, and eco-friendly characteristics. The use of such innovative biotechnologies would facilitate resource-efficient utilization, promote animal performance and health for sustainable development in livestock production.

**Graphical Abstract:**

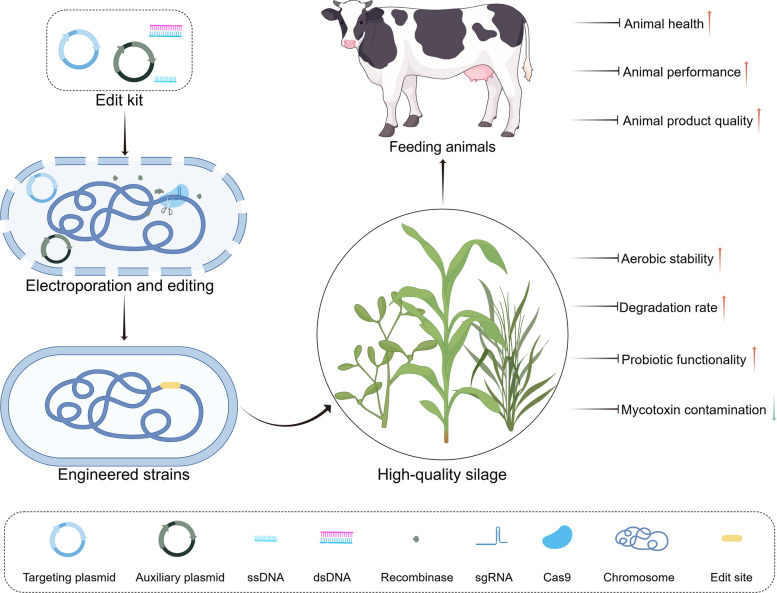

## Introduction

Livestock farming, as a crucial pillar of global agriculture, exerts profound impacts on human livelihoods. Livestock products provide readily bioavailable sources of protein, fat, micronutrients and other elements important for human health [1]. However, animal husbandry faces critical challenges including feed resource shortage, low feed conversion efficiency, and ecological issues such as grassland degradation. Hence, achieving sustainable development while ensuring food security has become an urgent global imperative.

To address the challenges associated with shortage in the supply of feed resources, forage conservation techniques, especially silage, are adopted. Silage is a high-quality biological feed produced via microbial fermentation of diverse plant materials such as pasture, forage crops and agricultural residues [2]. Silage enables long-term storage while maintaining the nutritional properties of the plant material, which makes it possible to provide a continuous supply of feed for livestock during seasons of low forage availability [3], ensuring stable livestock production. During fermentation, lactic acid bacteria (LAB) utilize soluble sugars from plant biomass to produce lactic acid and other beneficial organic acids to reduce pH of the ensiling environment, thus inhibiting the growth of spoilage microorganisms [2, 4]. Moreover, existing studies have demonstrated that inoculating functional LAB strains during ensiling not only upregulates the expression of fibrolytic enzymes to promote utilization efficiency of feed, but also boosts the production of beneficial bioactive metabolites for improved animal health [5, 6]. These innovations have substantially enhanced both the nutritional quality and economic viability of silage, while concurrently reducing the livestock's dependence on natural pastures. This synergistic effect enables the animal husbandry industry to realize win–win outcomes of resource conservation and production sustainability, thus improving the economic and practical value of feed.

Among the numerous microorganisms involved, *Lactobacillus* is the most widely studied and applied, attributed to its extremely high lactate production, acid tolerance, osmotic pressure tolerance, pro- and post-biotic properties. The enzymes and metabolites produced by *Lactobacillus* can directly affect the fermentation quality of silage. Many studies focus on using *Lactobacillus* inoculants to improve the fermentation process to ensure the quality of silage [7, 8]. However, the screening process for naturally occurring, high-performing *Lactobacillus* strains is time-consuming and labor-intensive. The advances in synthetic biology now enable functional gene introduction or direct genome editing in *Lactobacillus*, conferring either enhanced native traits or novel functionalities. When applied to silage, these engineered strains serve as both fermentation starters and performance enhancers. Their superior efficacy positions engineered *Lactobacillus* as next-generation inoculants. For example, alfalfa silage was inoculated with *L. plantarum* engineered to carry heterologous genes encoding cellulase (CbXyn10C) and xylanase (Bgxg1). This treatment resulted in significant reductions in hemicellulose (17%), cellulose (6%), and ADL (14%) compared with control, while increasing water-soluble sugar [9]. Beyond gene introduction via plasmid vector, precision genome editing enables direct modification of *Lactobacillus* chromosomal DNA to achieve stable target gene expression. Among contemporary editing tools, CRISPR-Cas is widely used to construct stable engineered strains due to its high efficiency, accuracy and scarlessness in editing [10–12]. In addition, the Cas9 (a Type II single-effector protein)-based CRISPR system prevails in current research and applications owing to its simplicity and cross-species portability [13, 14]. CRISPR-Cas9 technology has demonstrated preliminary success in knockout, insertion and mutation of gene fragments in *Lactobacillus* [14, 15]. For instance, the CRISPR-Cas9-assisted genome editing system enabled precise genetic modifications in *L. plantarum* WCFS1 [16]. Specifically, the *nagB* gene (whose product catalyzes the reverse reaction of L-glutamine-F6P aminotransferase) was knocked out using CRISPR-Cas9-assisted dsDNA recombination. Additionally, riboswitch replacement and point mutations were introduced into the *glmS1* gene (encoding L-glutamine-F6P aminotransferase) via double-stranded and single-stranded DNA (dsDNA and ssDNA) as repair donors to alleviate product feedback inhibition. These modifications enhanced the specific metabolic pathway, resulting in a N-acetylglucosamine yield of 797.3 mg/L, positioning the engineered strain as a promising candidate for industrial applications. These advances elucidate promising pathways for developing high-performance strains tailored for silage production and preservation. This review systematically analyzes and prospectively evaluates the feasibility of employing CRISPR-Cas9-based genome editing tools to construct optimized functional *Lactobacillus* strains, with particular emphasis on overcoming key challenges encountered in silage production.

## Silage as a cornerstone of livestock industry development

The ensiling process typically undergoes four distinct phases: (1) an aerobic phase, where oxygen is consumed by residual plant respiration and microbial activity; (2) a fermentation phase, where bacteria, primarily LAB, dominate and convert water-soluble carbohydrates (WSCs) into organic acids (mainly lactate) under anaerobic conditions, leading to a rapid pH drop; (3) a stable phase, where low pH and anaerobic conditions inhibit spoilage microorganisms, preserving the silage; and (4) a feed-out phase, where exposure to air risks aerobic spoilage [17]. Therefore, inoculating with LAB strains has become a key strategy to optimize silage fermentation and achieve excellent forage preservation.

Dubbed as “canned forage” [5], silage provides a consistent and nutritious feed for livestock during seasons when fresh pasture is scarce [3], while having a significant positive impact on livestock performance and health and playing a crucial role in modern animal husbandry systems.

### Efficient utilization of diverse substrates

Silage converts abundant human-inedible and non-conventional feedstuffs into valuable livestock feed, mitigating feed resource scarcity while alleviating human-livestock competition for grain. Traditional silage substrates primarily include forage cereals (e.g., whole-plant corn or sorghum) and forage grasses/legumes (e.g., alfalfa, oat hay or Chinese leymus). Amid growing feed resource constraints, lignocellulosic agricultural residues such as bagasse, spent mushroom substrate, and crop straws have been used as alternative feed ingredients [18–20]. The recalcitrant plant cell wall impedes nutrient digestion and absorption in animals [21]. Organic acids and enzymes produced by LAB during ensiling partially degrade lignin and other structural carbohydrates, disrupting plant cell wall architecture. This process enhances cellulose and hemicellulose accessibility, ultimately improving the degradability and digestibility of fibrous plant components [21].

The digestibility of silage is highly dependent on the effectiveness of silage additives. For recalcitrant non-conventional feedstuffs, enzymatic preparations and LAB inoculants can be employed to enhance fermentation quality. Exogenous fibrolytic enzymes are routinely supplemented during ensiling. Their synergistic interactions with LAB are demonstrated to reduce the acid detergent fiber (ADF) and neutral detergent fiber (NDF) contents while increasing the crude protein (CP), WSC levels in the silage [21, 22]. Moreover, LAB inoculants enhance the nutritional preservation rate and fiber degradation efficiency of silage through rapid acidification and enzyme-acid synergism [23, 24]. Notably, fibrolytic enzyme-producing LAB inoculants play a pivotal role in silage fermentation [25]. Their dual function of fermentation and enzyme production reduces the economic burden of exogenous enzyme supplementation. Compared to direct ensiling, LAB inoculation promotes the disintegration of recalcitrant structural polysaccharides (Table 1), thereby enhancing silage quality. This approach has emerged as a crucial methodology for ensiling non-conventional feed resources.

**Table 1:** Effects of lactic acid bacteria (LAB) inoculation on the reduction of fibrous components in silage compared to direct ensiling

Silage substrates	Additives	Silage days	Reduction of fibrous components	References
Rice straw	*L. plantarum*, *L. salivarius*, *L. reuteri*, *L. brevis* and *Streptococcus bovis*	30 d	NDF: 5.13%–8.69% ADF: 7.57%–13.03%	[18]
Rice straw	*L. plantarum*	60 d	NDF: 11.69% ADF: 9.06% Cellulose: 9.51% Hemicellulose: 7.85%	[24]
Potato + Wheat straw	*L. buchneri*, *L. plantarum*	90 d	NDF: 1.81%–2.72% ADF: 1.73%–3.90%	[26]
Wheat straw	*L. plantarum*	60 d	NDF: 9.15% ADF: 14.33% ADL: 15.40%	[27]
Sugarcane bagasse	*L. casei* + cellulase	30 d	NDF: 1.67%	[28]
Spent mushroom substrate	*L. plantarum*	45 d	ADF: 4.09%	[29]
Soybean residue + Corn stover	LABs + cellulase	56 d	NDF: 15.78% ADF: 17.20% ADL: 18.58% Cellulose: 16.77% Hemicellulose: 13.63%	[30]
Sawdust-based spent mushroom substrate	2 *L. plantarum* strains	10 d	ADF: 3.86%	[31]
Paper mulberry	*L. plantarum* + xylanase	60 d	NDF: 5.03% ADF: 4.80%	[32]
Paper mulberry	*L. plantarum*	60 d	NDF: 9.05%	[33]
Cauliflower leaf	*L. plantarum*	30 d	NDF: 6.60% ADF: 14.23% ADL: 16.67% Cellulose: 13.64%	[34]

*Reduction of fibrous components* Values represent the percentage reduction of a specific fibrous component in LAB-inoculated silage compared to direct ensiling (control), calculated as: (Control – LAB)/Control × 100%

*NDF* Neutral detergent fiber, *ADF* Acid detergent fiber, *ADL* Acid detergent lignin

### Enhancing animal performance and animal product quality

Silage exerts beneficial effects on ruminant production performance. Corn silage has emerged as the dominant forage component in the dairy cow ration over the last few decades [35]. Incorporating corn silage into dairy rations enhances dry matter intake (DMI), milk yield, and milk protein content [36]. A comprehensive analysis demonstrated that incorporating 51% corn silage into grass silage-based diets significantly improved milk composition in dairy cows, with notable increases in protein, fat, and lactose yields. The supplementation elevated milk production by 1.8 kg/d and enhanced milk protein concentration by 1.2 g/kg. Notably, ensiled corn with dry matter content ranging from 300 to 350 g/kg demonstrates progressive improvements in both milk yield and protein content of dairy cows as the crop advances in maturity [35]. Beyond corn silage, the addition of non-corn forage silage inoculated with homofermentative and facultative heterofermentative LAB into the feeding strategy exerts positive effects on milk yield, milk fat content, and milk protein concentration of dairy cows [37, 38]. In monogastric animals, silage feeding shapes porcine gut microbiota, improves their carcass traits and enhances pork quality [39, 40]. Similarly, feeding ducks and geese with cost-effective silage has a positive impact on production metrics (in terms of feed conversion, meat quality and edible offal weight) without compromising growth performance or meat nutritional composition [41, 42]. Collectively, high-quality silage-based animal production systems enhance farm sustainability.

### Promoting animal health

In livestock husbandry, feed should not merely satisfy their nutritional requirements, but also serve as delivery agent for beneficial bioactive compounds to promote health and welfare, which ultimately contributes to the production of high-quality livestock products. The silage process involves complex dynamic microbial succession and the quality of silage largely depends on evolving microbial community structure and their metabolic outputs. Various functional metabolites with anti-inflammatory, antioxidant and anticarcinogenic activities were found to be enriched in silage, which demonstrates its potential therapeutic value in enhancing livestock health performance. Dietary supplementation with paper mulberry silage enhances antioxidant status and immune competence in ruminants, potentially attributable to its abundant bioactive compounds including phenolic acids, flavonoids, and alkaloids [43]. Additionally, the application of LAB inoculants will further optimize the beneficial functions of silage. For instance, the inoculation of *L. plantarum* strains with high-antioxidant activity enhances the antioxidant capacity of the silage, likely due to the production/perservation of non-enzymatic antioxidants such as carotenoids, tocopherols, and flavonoids [44]. Feeding such silage helps mitigate oxidative damage in animals and improves their antioxidant status through modulating the expression of genes related to antioxidant defense and inflammation in the mammary gland, thereby ultimately enhancing milk quality [45]. In addition to beneficial compounds, silage fermentation effectively reduces the abundance of antibiotic resistance gene s (ARGs) and their pathogenic hosts. Studies demonstrate that inoculants like *L. plantarum* suppress harmful bacteria growth and minimize plasmid-mediated ARG transfer, thereby decreasing the enrichment of ARGs (e.g., vancomycin, aminoglycoside resistance genes) in silage [46, 47]. This mitigation of ARG dissemination through the feed-animal-food chain provides a crucial strategy for safeguarding animal health and food safety.

### Reducing mycotoxin contamination

Mycotoxins are toxic secondary metabolites produced by various fungi. Feed ingredients are susceptible to fungal (particularly mold) contamination during harvesting or storage, which can subsequently lead to mycotoxin production [48]. Consumption of feed contaminated with mycotoxins can adversely affect livestock, resulting in organ toxicity and damage, immunosuppression, reduced fertility, decreased productivity, and even death [49, 50]. This raises major feed safety issues in global agricultural production. Even more seriously, mycotoxin contamination in food chains threatens human health through secondary exposure. Ingested mycotoxins and their metabolites can remain in most animal tissues and products (e.g., meat and milk). The residual toxins can be transmitted to humans through the consumption of these animal tissues and products [50], posing serious health and hygiene risks. Silage reduces mycotoxin hazards in feeds. Diverse LAB strains, such as *L. plantarum*, are recognized to suppress the proliferation of yeast and spoilage molds, as well as the contamination of mycotoxin (e.g., AFB1 or OTA) during the ensiling process. These effects can be achieved through metabolite production, cell wall adsorption, or direct mycotoxin degradation [51, 52]. On the other hand, the addition of chemical and phytogenic additives into silage could simultaneously inhibit the reproduction of yeasts and moulds [53] and disrupt the biosynthetic pathways of mycotoxins [54], providing an effective strategy of controlling mycotoxin contamination of silage.

In summary, silage serves as a cornerstone of modern animal agriculture, providing a consistent, high-quality, and cost-effective feed source. It supports animal health, productivity, and agricultural sustainability, making it an indispensable feed resource for livestock producers.

## Challenges in silage production and application

Despite demonstrated benefits of silage, large-scale production faces multiple challenges.

### Inefficient fermentation

Intrinsic challenges during the silage fermentation process are non-negligible and often act as the primary cause of downstream problems. The production of lactate by LAB and the subsequent rapid pH drop during silage production are critical processes for inhibiting pathogenic bacteria and preserving the nutritional value of the feed [55]. However, the establishment of this process can be disrupted by various factors. First, silage fermentation relies on the combined activity of inoculants and epiphytic microorganisms; the speed and efficiency of fermentation can be hindered by interference from native epiphytic bacteria [55]. Second, silage quality is influenced by ambient temperature. High temperatures can weaken the growth and metabolism of LAB, leading to a heterolactic fermentation [56], whereas lower temperatures may reduce LAB growth, resulting in a slow acid production [57]. Furthermore, silage quality is also affected by the characteristics of the raw materials themselves. For instance, some materials exhibit high buffering capacity and low WSC content (e.g., alfalfa) [58], while others have high-moisture characteristics (e.g., oats) [59]. These factors can impede the rapid establishment of desirable fermentation patterns, providing an prolonged window for the proliferation of undesirable microorganisms such as clostridia, enterobacteria, yeasts, and molds [60]. This may lead to the production of harmful metabolites like ammonia and butyrate, as well as the occurrence of secondary fermentation, ultimately resulting in poor feed quality. Therefore, addressing these fermentation bottlenecks is important for producing high-quality silage that supports animal performance and health.

### Aerobic instability

Oxygen may infiltrate into silage during the storage or feed-out phase due to inadequate compaction, poor covering techniques, slow feed-out speeds or compromised plastic integrity, triggering the proliferation of aerobic microorganisms (e.g., yeasts, moulds, aerobic bacteria) and leading to secondary fermentation [61, 62]. Yeasts serve as the primary drivers of silage aerobic spoilage. They adversely affect aerobic stability when their populations exceed 1 × 10^5^ CFU/g fresh weight [63]. WSC-assimilating yeast metabolize WSC to produce ethanol, while lactate-assimilating yeast oxidize lactate to release CO₂ and heat, leading to pH increases and temperature fluctuations [63, 64]. The activity of aerobic microorganisms accelerates the depletion of fermentation products, increasing dry matter (DM) losses and reducing the nutritional value of silage. Furthermore, the proliferation of undesirable microorganisms may elevate the risk of pathogens and their metabolite contamination, negatively affecting animal performance and health. Consequently, suppressing aerobic microbial activity represents a core challenge in ensuring silage stability.

### Mycotoxin contamination

Feed ingredients are susceptible to fungal infestation during harvest or storage, leading to mycotoxin contamination [48]. Globally, numerous surveys have been conducted on mycotoxins in silage, with corn silage being the most extensively investigated [65, 66]. The analyzed corn silage samples generally showed severe mycotoxin contamination, attributable to suboptimal ensiling conditions, with high prevalence of deoxynivalenol (DON), zearalenone (ZEN), and fumonisins in most samples [65–67]. Although silage fermentation can suppress some fungal growth through acidic conditions, the metabolic activity of acid-tolerant microorganisms may still cause secondary toxin accumulation. More seriously, multiple mycotoxins often co-occur in symbiotic forms. Due to synergistic effects among different aflatoxins and other metabolites, the toxin combinations exhibit additive effects, with naturally occurring forms potentially causing more severe damage [68, 69]. Therefore, developing microbial inoculants with dual antifungal and detoxification capabilities is key to mitigating toxin risks in silage.

### Low feed degradation rates

Plant materials possess rigid cell walls, where polysaccharides such as cellulose, hemicellulose, pectin, and lignin are intricately interwoven, conferring significant physical, chemical, and microbial resistance [70]. The presence of phenylpropane polymer lignin constitutes the primary barrier to cell wall deconstruction. This not only forms a recalcitrant polysaccharide fortress but also creates steric hindrance through ferulic and coumaric acid cross-linkages with other polysaccharides [71, 72]. During ensiling, acids along with plant- and microbial-derived polysaccharidases synergistically degrade plant cell walls, disrupting the robust structure and improving digestibility of the plant material. Nevertheless, a substantial portion of lignocellulose remains intact, particularly in highly lignified materials [73], such as late-harvest forage crops and crop stalks. These materials exhibit poor ensiling performance and low feed digestibility, resulting in resource underutilization. Thus, more effective methods are required to liberate cell wall sugars to enhance livestock utilization of silage.

### Insufficient probiotic functionality

Silage serves not only as a nutritional vehicle but also as a delivery system for prebiotics (e.g., oligosaccharides), probiotics (e.g., *Lactobacillus*), and postbiotics (e.g., bacteriocins). It modulates animal metabolism and immunity via the gut-organs axis. Researchers have identified native *Lactobacillus* strains producing bioactive compounds, including bacteriocins, γ-aminobutyric acid (GABA), exopolysaccharides (EPS), and indole-3-lactic acid (ILA) [74–77]. These compounds were demonstrated to have high efficacy in enhancing immune competence, growth performance, and overall health.

Specifically, bacteriocins selectively inhibit pathogenic bacteria (*Listeria monocytogenes*, *Escherichia coli*, etc.) [78], maintaining gut microbiota balance to reduce infections and antibiotic dependence. As a neurotransmitter, GABA alleviates stress responses, improves feed intake and growth performance, while modulating immune function of animals [79, 80]. The EPS strengthens the intestinal mucus barrier, promotes probiotic colonization, and regulates immune cell activity to enhance disease resistance [81]. ILA, a tryptophan metabolite, activates the aryl hydrocarbon receptor (AhR), mitigates intestinal inflammation, reinforces epithelial barrier integrity, and stimulates short-chain fatty acid (SCFA) production to optimize energy metabolism [82, 83]. However, studies on these functional *Lactobacillus* strains in silage and their impacts on livestock remain limited. Greater research efforts should focus on isolating and enhancing functional *Lactobacillus* strains for silage applications.

## Strategies and limitations for improving silage quality

Chemical and biological additives serve as effective approaches to enhance fermentation and optimize silage quality. However, the application of chemical additives is constrained by safety concerns and economic costs. In contrast, functional microbial inoculants can simultaneously improve fermentation while producing beneficial enzymes or metabolites, offering a simple, cost-effective, and efficient way for high-quality silage production.

To address the challenges in the silage fermentation process, increased research has focused on screening highly efficient LAB strains to facilitate rapid establishment of fermentation. For example, high temperature at 45 °C resulted in poor fermentation of corn silage, whereas the addition of thermotolerant *L. rhamnosus* LR753 led to the highest LAB population and improved fermentation quality [84]. Under low-temperature conditions, a study by Su et al. [85] demonstrated that inoculation with exopolysaccharide-producing *L. plantarum* L75 in oats ensiled at 15 °C for 60 days yielded the lowest pH value, the highest lactate content, and the lowest ammonia-nitrogen (NH₃-N) concentration. Furthermore, when silage materials such as alfalfa or high-moisture crops exhibit inherently poor ensiling potential, inoculation with *Lactobacillus* strains significantly enhances silage quality, markedly increasing lactate production while reducing pH and fiber content (NDF and ADF) [59, 60].

For controlling fungal and mycotoxin contamination, LAB inoculants also represent an effective strategy. Firstly, the addition of heterofermentative LAB, particularly *L. buchneri*, leads to the production of substantial acetate during ensiling, which suppresses the growth of molds and yeasts and improves the aerobic stability of silage [86]. Beyond organic acids, numerous LAB strains capable of producing potent antifungal compounds have been screened. For example, *L. plantarum* MiLAB 14 produces 3-(R)-hydroxydecanoic acid, 3-hydroxy-5-*cis*-dodecenoic acid, 3-(R)-hydroxydodecanoic acid, and 3-(R)-hydroxytetradecanoic acid, which significantly inhibit the growth of several molds and yeasts [87]. *L. plantarum* MiLAB 393, isolated from silage, is capable of synthesizing antifungal cyclic dipeptides, including Cyclo(L-Phe-L-Pro) and Cyclo(L-Phe-*trans*-4-OH-L-Pro), as well as 3-phenyllactic acid [88]. Furthermore, certain LAB strains demonstrate the ability to adsorb and biodegrade mycotoxins, thereby reducing their toxicity [89].

To overcome lignocellulose recalcitrance in silage, microbial strains producing fibrolytic enzymes (e.g., feruloyl esterases [25], cellulases [90]) have been screened to enhance plant carbohydrate availability. Additionally, probiotic bioactive compounds in silage can enhance anti-inflammatory and antioxidant capacities, thereby optimizing rumen microbial function and animal health. An increasing number of strains producing these functional metabolites have been isolated, showing promise for developing value-added silage [91, 92]. Beyond functional strain screening, composite LAB inoculants have been employed to optimize fermentation profiles through a complementary and synergistic effect. The strategy enables more balanced organic acid production and improved stability [93, 94].

To systematically clarify the core challenges in silage production and the preliminary strategies to address them, the key points summarized above are organized in Table 2. Although functional strain selection and multi-strain interactions have demonstrated efficacy in silage enhancement, the screening process remains labor-intensive and time-consuming. Key challenges include: (1) the extremely low abundance of natural ideal strains, with non-targeted screening strategies lead to a high degree of randomness in obtaining target strains; (2) frequent functional limitations (e.g., single function, poor environmental adaptability) in candidate strains; and (3) substrate-dependent performance variability of LAB consortia, hindering broad-spectrum and efficient silage improvement.

**Table 2:** Key challenges in silage production and corresponding mitigation strategies

Challenge	Root causes	Consequences	Feasible strategies
Inefficient fermentation	Interference from epiphytic microbes; unsuitable ambient temperature; unfavorable characteristics of raw materials	Proliferation of undesirable microorganisms; production of harmful metabolites; secondary fermentation	Inoculation with highly efficient LAB strains adapted to various adverse conditions
Aerobic instability	Oxygen infiltration	Proliferation of aerobic microorganisms; aerobic spoilage	Inoculation with heterofermentative LAB; screening of LAB strains capable of producing antifungal compounds
Mycotoxin contamination	Fungal infestation	Mycotoxins production and accumulation	Application of LAB strains with antifungal and mycotoxin-detoxification capabilities
Low feed degradation rate	Recalcitrance of plant cell wall components	Poor ensiling performance and low feed digestibility	Supplementation with fibrolytic enzymes-producing LAB strains
Insufficient probiotic functionality	Limited studies on functional *Lactobacillus* strains with probiotic functionality in silage	Failure to confer health benefits through silage	Screening of LAB strains producing probiotic bioactive compounds

## Engineered *Lactobacillus*: a synthetic biology strategy for silage improvement

**Fig. 1:**
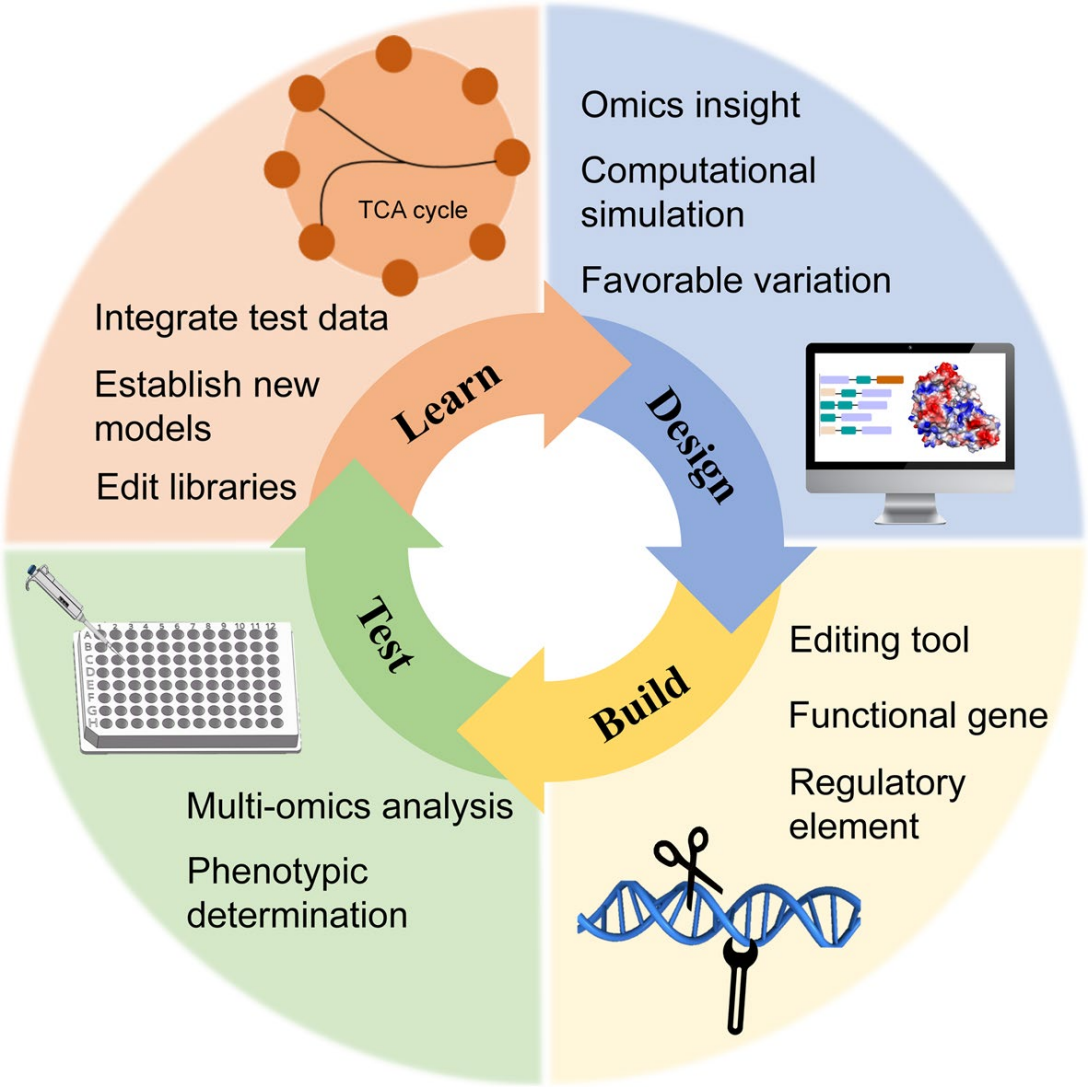
Design-Build-Test-Learn workflow for cell factory development and implementation. This workflow is applicable to the development of efficient *Lactobacillus* strains discussed in this review. Select target traits and optimal gene-editing strategies (Design). Rapidly generate mutant libraries via gene-editing tools (Build), significantly accelerating strain development compared to lengthy natural selection. High-throughput screening based on phenotypic metrics and sequencing data to identify ideal candidates (Test), and computational integration of the information set of beneficial mutations will guide the next design cycle (Learn)

Advances in synthetic biology have introduced a new era of microbial resource development, enabling the precise engineering of microorganisms for diverse biotechnological applications. Employing the Design-Build-Test-Learn (DBTL) cycle [95] (Fig. 1), synthetic biology enables rapid development of new biological systems and engineered strains through iterative optimization for specific functions and applications. This iterative model dramatically improves microbial engineering efficiency and success rates by guiding strain design via computational simulations, validating performance through high-throughput screening, and optimizing predictive models with experimental data. Each iteration yields superior engineered strains and accumulates valuable knowledge for future research. Such engineering strategies have profound applicability and they not only facilitate the discovery of novel functional genes and bioactive compounds but also create novel microbial resources with high productivity, robust adaptability, and enhanced activity. These optimized microorganisms have tremendous potential to advance sustainable development and productivity of agriculture, thereby providing robust scientific and technological support for modern agricultural biotechnology industries.

Engineered strains with specific functionality can be constructed by introducing recombinant plasmids carrying exogeneous genes into the host bacteria. For instance, transforming LAB with plasmids encoding glycoside hydrolases and inoculating them into silage can significantly reduce fiber content and improve fermentation quality [9, 96]. The heterologous proteins can be expressed not only in the cytoplasm but also anchored to microbial surfaces via covalent or non-covalent attachment. The cell surface display technology achieved through the lipoprotein anchor or cell wall anchor pathway has successfully immobilized functional β-mannanase and chitosanase on the surface of *L. plantarum* and exerted their effects in oligosaccharide production [97, 98]. Plasmid transformation provides a straightforward approach for heterologous gene expression in *Lactobacillus*, facilitating both silage biomass degradation and potential prebiotic oligosaccharide production. This supports the feasibility of engineered *Lactobacillus* as a whole-cell biocatalysts for producing high-quality silage with beneficial effects. However, transformed plasmids are at risk of genetic loss and generally require the selective pressure of antibiotics [99]. Antibiotics may disrupt the balance of beneficial microbial communities during fermentation, interfering with the natural LAB-dominated fermentation process. Moreover, antibiotic residues could enter animal products and the environment through the feed chain, exacerbating public health risks such as bacterial resistance. To ensure stable gene expression while eliminating antibiotic selection, integration of target genes into the genome via gene-editing technologies represents a viable solution.

### Conventional genome editing technologies in *Lactobacillus*

Traditional genome editing technologies relying on zinc-finger nucleases (ZFNs) and transcription activator-like effector nucleases (TALENs) pioneered the genome editing era, enabling targeted gene deletions, insertions, and mutations at specific genomic loci. Both systems consist of sequence-specific DNA-binding domains fused to non-specific FokI nuclease, forming a dimer that generates double-strand breaks (DSBs) at target sites. This triggers cellular DNA repair pathways based on non-homologous end joining (NHEJ) or homology-directed repair (HDR) to achieve precise genome modifications [100, 101]. However, their practical application in *Lactobacillus* remains limited.

Additionally, integrative suicide plasmid vector supporting the double-crossover homologous recombination (HR) method enables insertion, deletion, and replacement editing in the genome. A suicide plasmid is used to integrate exogenous DNA fragments into the target gene locus through two HR events. During the first HR event, the entire plasmid integrates into the genome via recombination at one homologous arm. Subsequently, a second HR event occurs at the other homologous arm, which leads to the excision of the plasmid backbone and the precise incorporation of the exogenous DNA. This two-step chromosomal integration procedure has allowed researchers to stably integrate α-amylase [102] and endo-1,4-β-glucanase [103] genes into the genome of *L. plantarum* isolated from silage, without leaving any residual resistance markers or vector sequences. These genetically modified organisms are expected to enhance the carbohydrate utilization efficiency and acidification efficiency of the *Lactobacillus* strains in silage. However, the double-crossover homologous recombination method is cumbersome, time-consuming, typically requiring 1–2 weeks to obtain positive clones.

An alternative genome editing method is to utilize linear nucleotide chains as substrates to enable genetic engineering through HR in bacterial cells. The linear nucleotide chains include dsDNA and ssDNA. The recombination process is mediated by bacteriophage-origined recombinase systems, specifically the Red/RecET systems. The λ phage Red system consists of three key proteins: Gam, Exo, and Bet, while the Rac phage RecET system comprises RecE and RecT proteins. These components function synergistically to facilitate efficient genetic modification.

Specifically, the Gam protein inhibits host RecBCD and SbcCD nucleases, thereby protecting exogenous dsDNA from degradation. The 5'→3' exonuclease activity of Exo or RecE processes dsDNA to generate 3' ssDNA overhangs. The ssDNA-binding proteins Bet or RecT coat the generated ssDNA, preventing it from being degraded by host nucleases while promoting HR activity [104, 105]. Yang et al. [105] identified the Gam, Bet and Exo analogues encoded by the lp_0640-0642 genes in the genome of *L. plantarum* WCFS1 through bioinformatics analysis. They subsequently constructed an editing system based on dsDNA homologous recombination, successfully achieving both gene knockout and insertion in *L. plantarum* WCFS1. However, the chloramphenicol resistance gene was employed as a screening marker in this experiment. Following recombination, the resistance marker needed to be excised from the genome using Cre recombinase, which would leave a 34 bp residual sequence. Therefore, there remains a need to develop more efficient traceless editing tools to accomplish precise genomic modifications.

### CRISPR-Cas9-driven engineering of *Lactobacillus*

CRISPR-Cas technology is a revolutionary gene-editing technology that is more feasible, efficient, and precise [106]. The CRISPR-Cas system is widely present in *Lactobacillus* genomes and shows diversity [107]. Among the numerous editing tools, Cas9 (the single effector protein of the Type II CRISPR-Cas system) has gained prominence as a highly efficient genome-editing tool due to its simplicity and portability for heterologous expression on plasmids in multiple hosts. Thus, CRISPR-Cas9 has become the extensively studied and deeply explored system to date [14, 108]. CRISPR-Cas9 is a two-component system consisting of a guide RNA (gRNA) and the Cas9 nuclease. The gRNA directs specific recognition of both the protospacer adjacent motif (PAM) in the target DNA and the targeted complementary sequence, while the Cas9 nuclease introduces a double-strand break (DSB) at this site [109]. In prokaryotic cells, the resulting DSB can be repaired through homology-directed repair (HDR) using an exogenous homologous template as a donor, thereby generating the desired DNA changes [109].

The three CRISPR-Cas9-based gene-editing templates exhibit distinct characteristics in terms of editing efficiency, application scenarios, and technical complexity, with a comparative analysis as follows (Table 3).

**Table 3:** Comparison of application scenarios and advantages/disadvantages among three CRISPR-Cas9-based gene editing templates

Template type	Application scenarios	Advantages	Disadvantages
Plasmid repair template	Applicable to precise insertion, deletion, replacement and point mutation	① Broad application scope; ② Capable of large-fragment editing (> 1 kb); ③ High efficiency, with further enhancement with the assistance of recombinase	Requires construction of plasmids containing Cas9, sgRNA, and repair templates, with a relatively complex construction process
dsDNA repair template	Applicable to medium-fragment editing (100 bp–1 kb), including applications such as gene knockout and promoter replacement	dsDNA can be directly synthesized or amplified by PCR reaction. The acquisition method is relatively flexible	① Depends on recombinase systems (e.g., RecA/RecET), with low efficiency in some bacterial strains; ② dsDNA is highly susceptible to degradation in bacterial cells
ssDNA repair template	Applicable to short-fragment editing (< 100 bp), such as SNP mutations, small fragment insertions/deletions	① ssDNA can be directly synthesized and used without requiring additional plasmid construction; ② Low-cost synthesis of short ssDNA; ③ For saturation mutagenesis experiments, it outperforms both plasmid-based and dsDNA repair templates in terms of cost-effectiveness and operational convenience	① Depends on recombinase systems (e.g., RecA/RecET), with low efficiency in some bacterial strains; ② ssDNA is highly susceptible to degradation in bacterial cells

#### Plasmid repair template

**Fig. 2:**
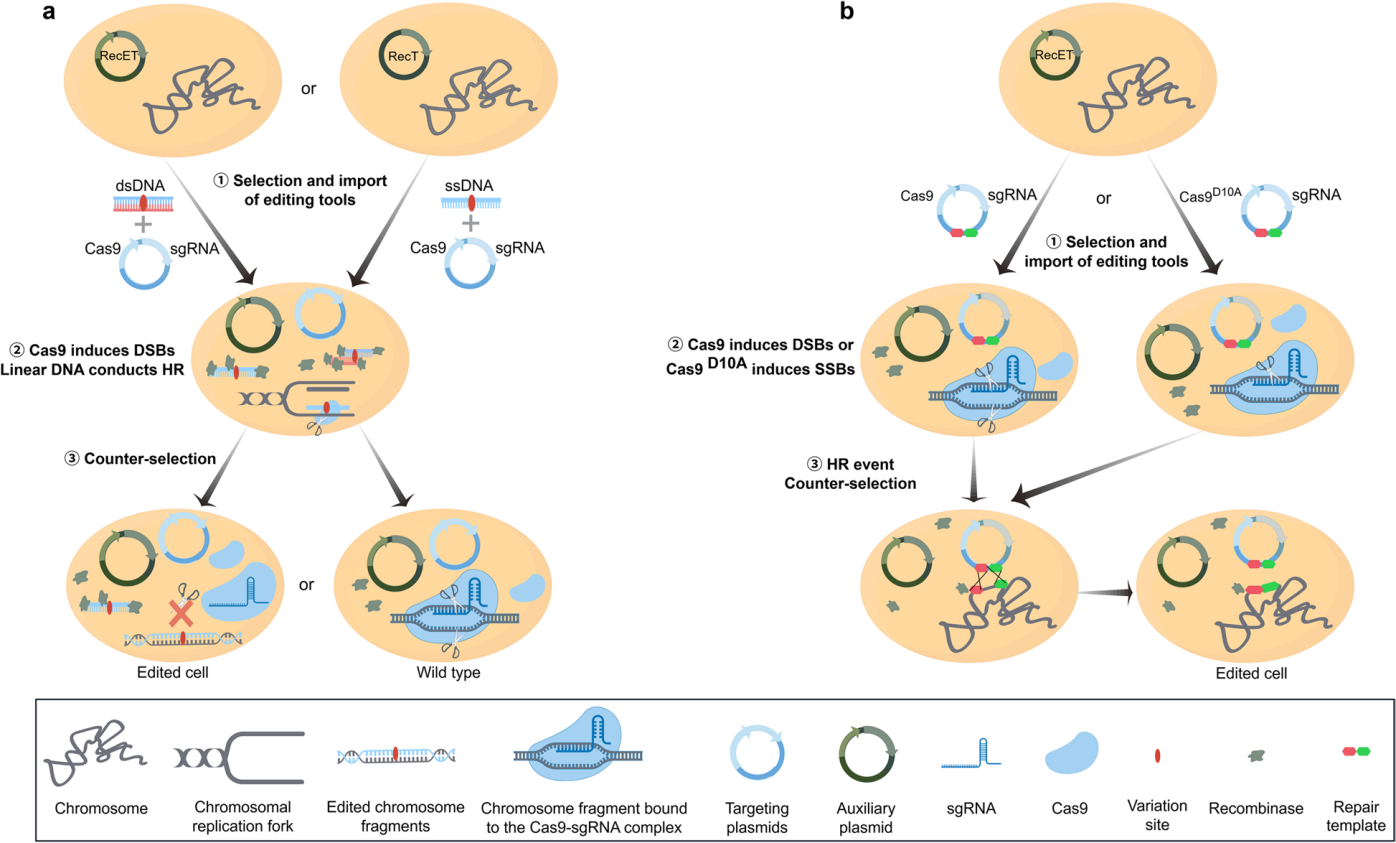
Genome editing of *Lactobacillus* using CRISPR-Cas9 system. **a** Under the assistance of recombinant enzymes, the CRIPSR-Cas9 system and linear DNA carrying variation sites were used to edit the genome of *Lactobacillus*. **b** Under the assistance of recombinant enzymes, the CRISRP-Cas9 system and targeted repair plasmids carrying variation sites were used to edit the genome of *Lactobacillus*

As shown in Fig. 2, during gene editing of *Lactobacillus* using the CRISPR-Cas9 system with repair plasmid-encoded homologous recombination templates, the Cas9 nuclease first induces a DSB at the target site, triggering the host cell's DNA damage response mechanism. This activates a series of signaling pathways that detect the DSB presence. The cell then utilizes the homologous recombination template encoded by the repair plasmid to achieve precise gene editing through the HR pathway. Furthermore, sustained expression of Cas9 nuclease serves as an effective negative selection strategy. Cells that fail to undergo successful editing cannot repair the DSB via HR pathway, leading to their selective elimination from the cell population. This significantly enhances the enrichment efficiency of the edited strains.

The use of CRISPR-Cas9-assisted repair plasmid-dependent editing generated an approximately 1,000 bp gene deletion in *L. plantarum* WJL [110]. Notably, some *Lactobacillus* strains fail to efficiently process Cas9/sgRNA complex-induced DSBs via native HR events even in the presence of repair donors. For such strains, host-specific RecE/T recombinases can be introduced to enhance HR efficiency. In *L. brevis* ATCC367, the assistance of endogenous RecE/T increased the editing efficiency of targeted repair plasmids by approximately 6- to 10-fold [15]. For strains with inherently low HR capability, the aforementioned methods still fail to enable them to escape DSB-induced death. In such cases, Cas9 variants (e.g., D10A nickase) represent an ideal alternative. Unlike DSBs, nickase-induced single-strand breaks (nicks) in chromosomal DNA can trigger HR while significantly improving bacterial survival rates. The targeted repair plasmid pLCNICK carrying the nickase was developed and reported by Song et al. [111]. Key elements in pLCNICK include the P_23_-Cas9^D10A^ and P_ldh_-sgRNA expression cassettes, along with homologous arms of the target gene serving as repair templates. Using pLCNICK and its variants, researchers achieved rapid and efficient deletion and insertion of chromosomal fragments in *L. casei* and *L. paracasei* [99, 111–113]. Similarly, the pLbCas9N plasmid contains the P6-SpyCas9^D10A^ and P_tuf_-sgRNA expression cassettes and the homologous arms of the target site as repair templates. It has been employed to establish gene deletion/insertion methods in *L. acidophilus* and *L. paracasei*, achieving remarkably high success rates (up to 100%) for gene deletions [114]. These portable tools establish a valuable platform for constructing a genetic editing toolbox for LAB.

#### dsDNA repair template

Compared to the Cre recombinase system, CRISPR-Cas9-based gene editing strategies enable the editing process using dsDNA as the repair template to be independent of resistance gene markers and leave no residual sequences in the genome, demonstrating superior editing characteristics. Taking *L. plantarum* WCFS1 as the research subject, the researchers developed an efficient CRISPR-Cas9-based gene editing strategy. The induced expression of the Red/RecET recombinase system in the auxiliary plasmid promotes precise replacement of target genes with dsDNA editing templates carrying homologous arms. Simultaneously, the Cas9 nuclease expressed by the CRISPR-Cas9 plasmid generates DSBs at the target site, which not only enhances HR efficiency but also causes the wild-type cells that have not been successfully recombined to die due to the failure of DSB repair, thereby enabling negative selection against unedited cells. The strategy successfully achieved marker-free and scarless knockout of target genes in *L. plantarum* WCFS1, with a positive rate of 53.3% [16].

#### ssDNA repair template

The ssDNA annealing protein can mediate bacterial genome editing using ssDNA (Fig. 2). The researchers proposed that the annealing protein catalyzes the annealing of ssDNA with a complementary single-stranded region near the DNA replication fork. Subsequently, ssDNA, as a primer, integrates variations into the nascent strand through DNA polymerase- and ligase-mediated replication [115, 116]. Unlike CRISPR-Cas9-assisted dsDNA recombination, the ssDNA-based approach only requires the RecT protein expressed by the auxiliary plasmid to protect and guide the ssDNA fragment for recombination. The Cas9 protein and gRNA complex, expressed from a targeted plasmid, can still serve as a counter-selection tool to eliminate strains that fail to undergo ssDNA-mediated recombination. These toolkits have been applied to the gene editing in *Lactobacillus*, achieving a remarkable 90%–100% success rate in introducing point mutations into the genomes of *L. reuteri* 6475 and *L. plantarum* WCFS1 [110, 117]. Surprisingly, even short 80-polymer ssDNA enabled gene fragment deletions. They successfully introduced 501 bp and 1,002 bp deletions into *L. reuteri* 6475 with efficiencies of 30% and 7%, respectively, with the assistance of CRISPR-Cas9 tool [117].

## The potential applications of CRISPR-Cas9 in optimizing *Lactobacillus* for silage improvement

**Fig. 3:**
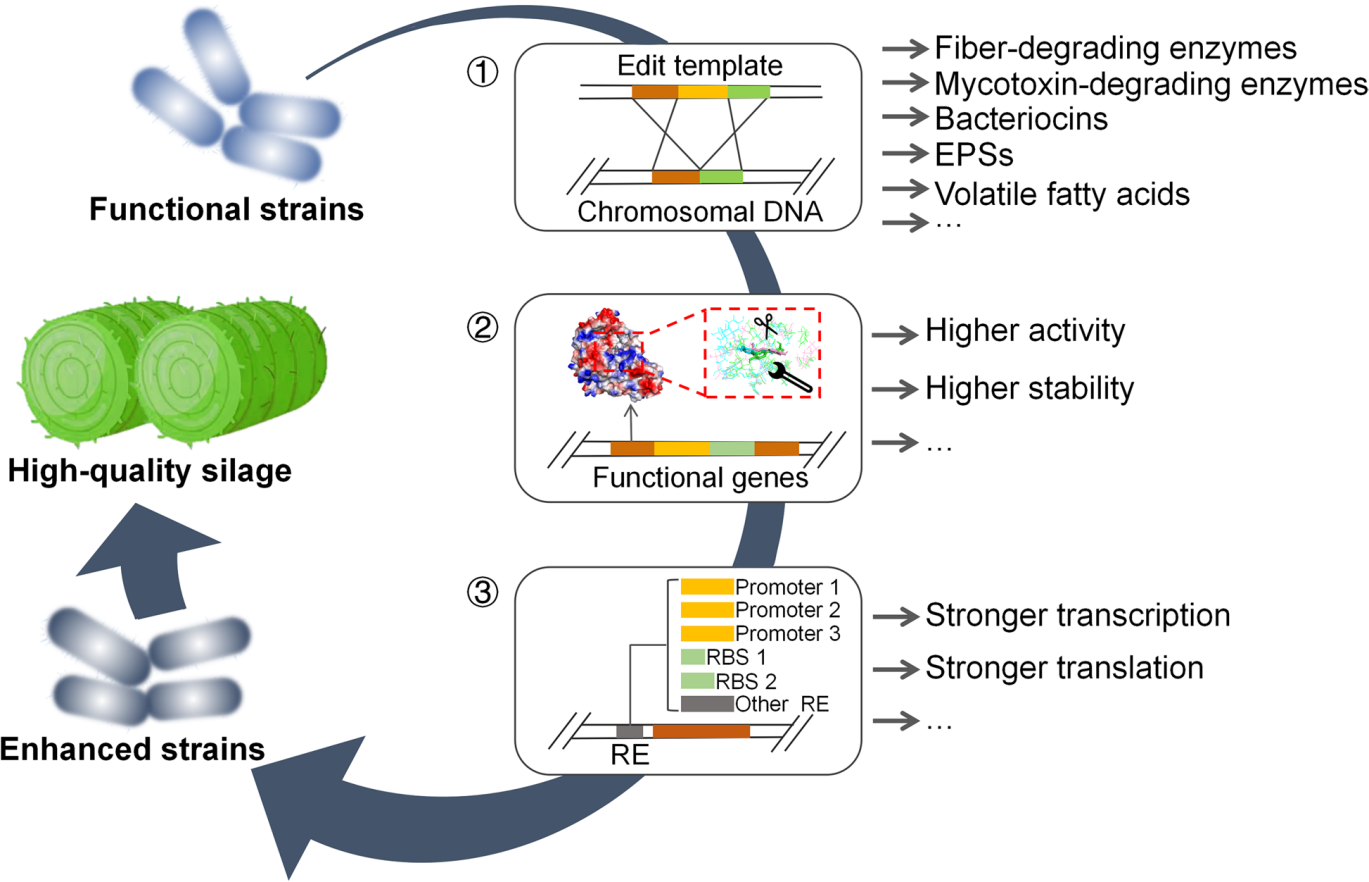
Potential applications of gene editing technology in optimizing *Lactobacillus* for silage improvement. ① Insertion of high-performance enzyme-coding genes into the *Lactobacillus* genome. Red and green segments represent homologous arms and the yellow denotes inserted genes (e.g., genes encoding fiber-degrading enzymes, mycotoxin-detoxifying enzymes, or enzymes related to the production of various metabolites). These enzymes/metabolites reduce mold proliferation and mycotoxin contamination while enhancing silage digestibility, aerobic stability, and probiotic properties. ② Engineering of inherent enzymes for functional enhancement. Focuses on improving enzymatic activity and stability. Red, green, and yellow segments indicate distinct functional genes. ③ Modification of regulatory elements to boost enzyme expression. Red segments indicate target gene and the gray indicate regulatory elements (RE)

Emerging genetic tools now offer new possibilities to overcome the limitations of traditional strains. Advanced genomic sequencing technologies have accelerated the discovery of metabolic mechanisms and functional traits in *Lactobacillus* strains, while genetic manipulation enables the introduction of novel characteristics or enhancement of inherent functions to the strains. This allows the engineering of strains with capabilities surpassing those of wild-type counterparts, ultimately used for improving silage process (Fig. 3).

### Enzyme introduction

The initiation of ensiling requires rapid lactic acid production by *Lactobacillus* to reduce the pH of the ensiling environment. Most *Lactobacillus* species are capable of producing both L- and D-lactic acid. Excessive intake of D-lactic acid can induce inflammatory responses and neurotoxicity in organisms [118, 119]. Tian et al. [112] obtained a *L. paracasei* strain for L-lactic acid production with high productivity (7.5 g/L/h) and high purity (exceeded 99.1%) by replacing ldhD with ldhL1 through a targeted repair plasmid relying on CRISPR-Cas9^D10A^ assistant. The degradation rate of ensiled plant materials is closely related to the digestibility of livestock. Previous studies added purified or commercial fibre-degrading enzymes to improve silage degradation [21, 22], which undoubtedly increased the cost for silage. The introduction of encoding genes of exogenous fibre-degrading enzymes is an economical and feasible approach. In addition to fibre-degrading enzymes, mycotoxin-degrading enzymes should also be introduced to remove the toxicity of silage. It has been reported that several microbial-derived redox enzymes [120], transferases [121], hydrolases [122], and fusion enzymes [123] are capable of degrading various types of mycotoxins. For *Lactobacillus* strains that cannot naturally secrete specific functional enzymes, the introduction of exogenous enzyme-encoding genes will enable them to acquire the non-native capability of enzyme secretion. The advancement of sequencing and high-throughput screening technologies will enable the discovery of an increasing number of functional enzymes, while the highly efficient CRISPR-Cas9 gene-editing system will pave the way for their integration. In addition to the aforementioned functional enzymes, some bacteria can produce bioactive compounds with antimicrobial and anti-inflammatory properties, such as EPS, bacteriocins, GABA, and phenyllactic acid (PLA). These substances can inhibit the growth of pathogenic bacteria in silage and promote livestock health. The introduction of genes associated with the biosynthesis of these bioactive substances will further enhance the probiotic properties of silage.

### Enzyme engineering

The application of functional bacterial strains producing specific enzymes has significantly improved silage quality. However, the recalcitrance of raw silage materials and instability of ensiling effects require further enhancement of the functional strains' efficacy, which can be achieved by boosting the activity of specific enzymes in the strains. Point mutations in enzymes can be employed to improve their performance, and this process can be computer-aided designed.

The evolving computer algorithms enables reliable prediction of enzyme crystal structures based on amino acid sequences. Leveraging structural information, the algorithms provide insights into enzyme-substrate binding, conformational changes, and reaction rates through molecular docking, molecular dynamics (MD) simulations, and quantum mechanical analyses. Furthermore, they predict favorable mutations by estimating parameters such as free energy changes [124]. Computer-assisted site-directed mutagenesis has been widely implemented for enzyme performance enhancement. Primarily, high catalytic activity requires optimal affinity and fitness between the substrate-binding pocket and its substrate. Computer-aided structural predictions and molecular docking guide the mutation of non-conserved residues in the active pocket to reshape its geometry. A resulting more open conformation, enhanced hydrophobicity, and reduced steric hindrance collectively improve substrate accessibility and stability, which leads to a higher catalytic efficiency [125]. Secondly, the flexibility of the enzyme conformation is recognized as critical for its stability. MD simulations can characterize protein unfolding dynamics at atomic-level resolution, providing insights that facilitate the modulation of local flexibility and rigidity to enhance protein stability [126]. Furthermore, thermostability prediction tools like PoPMuSiC can guide specific residue mutations to improve enzyme thermal stability by predicting the changes in folding free energy resulting from single-point substitutions [127]. Consequently, point mutations for enhanced enzyme performance can be obtained by computer algorithms and in vitro validation. CRISPR-Cas9-assisted linear DNA recombination technology enables direct integration of these verified beneficial mutations into specific chromosomal loci of functional bacterial strains, facilitating the acquisition of high-efficacy variants.

### Regulatory element (RE) optimization

Changes in some REs might enhance the function of *Lactobacillus* strains by modulating the level of gene expression.

#### Promoter optimization

Primarily, as the most critical RE for gene expression, appropriate promoter significantly enhances transcription of target genes [128]. Strong constitutive promoters facilitate stable and sustained overexpression of enzymes in *Lactobacillus* strains, which is particularly advantageous for their performance in large-scale fermentation processes (e.g., silage production). Numerous promoters have currently been identified from *Lactobacillus* strains, with their transcriptional activities evaluated across multiple *Lactobacillus* species (Table 4). For instance, in *L. plantarum* WCFS1, promoters P11 and P_tuf33_ demonstrate higher transcriptional activity compared to the L-lactate dehydrogenase promoter P_lp_0537_, whereas in *L. brevis* ATCC367, P11 and P_slpA_ exhibit superior activity over P_tuf33_ [15]. Notably, some heterologous promoters derived from other bacterial species display cross-species activity in *Lactobacillus*, e.g. promoters from *Bacillus subtilis* and *Enterococcus* were active in multiple *Lactobacillus* species [129, 135].

**Table 4:** List of efficient constitutive promoters commonly used in *Lactobacillus*

Promoter name	Squence source	Strain source	Applied strain	Plasmid backbone	References
P_lp_0537_	L-Lactate dehydrogenase	*L. plantarum* WCFS1	*L. plantarum*	pLCNICK	[15]
P_pgm_	Phosphoglycerate mutase	*L. acidophilus* NCFM	*L. plantarum*	pSIP409	[97]
P_ldh_	Lactate dehydrogenase	*L. acidophilus*	*Lactococcus lactis, L. reuteri*	pTRKH3	[129]
P_slp_	Surface layer protein	*L. acidophilus*	*Lactococcus lactis, L. reuteri*	pTRKH3	[129]
P_ermB_	rRNA adenine N-6-methyltransferase	*Enterococcus faecalis*	*Lactococcus lactis, L. reuteri*	pTRKH3	[129]
P11	rRNA	*L. plantarum* WCFS1	*L. plantarum* and* L. sakei*	pSIP409	[130]
P_tuf33_	Elongation factor	*L. plantarum* CD033	*L. plantarum*	pCDLbu-1	[131]
P_tuf34_	Elongation factor	*L. buchneri* CD034	*L. plantarum*	pCDLbu-1	[131]
P_slpA_	Surface layer protein	*L. brevis, L. acidophilus* ATCC4356	*Lactococcus lactis, L. plantarum* and* L. gasseri*	pKTH2095, pSIP409	[97, 132]
P_ldhL_	L-Lactate dehydrogenase	*L. sakei*	*L. delbrueckii* ssp*. lactis*	pLEM415	[133]
P_23_	Uncharacterized gene	*Lactococcus lactis*	*L. plantarum, Lactococcus lactis*	pLCNICK, pOri253	[15, 134]

In addition to natural promoters, synthetic promoters are used to optimize production. Rud et al. [130] constructed a library of constitutive synthetic promoters by randomizing the non-consensus spacer sequences in rRNA promoters within the *L. plantarum* WCFS1 genome. The resulting potent synthetic promoters enable stable and efficient protein production. Similarly, Zhang et al. [136] mutated the −35 and −10 regions of a promoter of the expression plasmid in the same strain to match consensus sequences. This modification significantly improved promoter activity, increasing the relative fluorescence units by up to 12.7-fold. Except changes in promoter sequences, the number of promoters also has a significant impact on gene expression. Constructing repeat promoters may be an effective strategy to enhance the expression intensity of target genes, as has been practiced in other bacteria [137]. Therefore, using gene-editing techniques to directly replace or mutate the original promoter, or increase the number of promoters of the target gene, is expected to enhance gene overexpression.

#### Ribosome binding site (RBS) optimization

Secondly, RBS controls the efficiency of mRNA translation initiation, strongly affecting protein production levels [131]. Fine-tuning the composition of the RBS sequence, or the distance between the RBS sequence and the starting codon, will result in different translation levels. By substituting the RBS sequence in the *L. plantarum* WCFS1 expression plasmid's PlacA promoter with "AGGAG", a closer match to the bacterial consensus RBS "AGGAGG" and nearer to the start codon, researchers achieved enhanced RBS strength. This optimization increased relative fluorescence units by 1.6-fold [136]. Similarly, Tauer et al. [131] reported that in the expression plasmid of *L. plantarum*, changes in the sequence of Shine-Dalgarno and its distance from the translation initiation site result in adjustable translation efficiency. Altering the RBS may affect translation initiation rate and subsequent translation efficiency by changing the average ribosomal distance [138]. Therefore, through gene-editing techniques, the RBS sequence before the target protein-encoding gene can be altered to change the translation levels of the proteins, thereby regulating metabolic flux and obtaining corresponding metabolites.

## Future perspectives

### CRISPR-empowered engineering of functional *Lactobacillus* promotes silage quality and functionality for sustainable livestock development

Against the backdrop of multiple challenges such as resource constraints, antibiotic abuse and environmental pollution faced by the global livestock industry, silage, as a crucial nutritional source for animals, its quality directly impacting animal health and production performance. However, traditional ensiling processes suffer from issues such as low fermentation efficiency, harmful microorganism proliferation, and nutrient losses, which urgently demand innovative technological solutions. The rapid advancement of CRISPR gene-editing technology provides a powerful tool for precise modification of functional *Lactobacillus*. To translate this technical potential into practical applications, future research should prioritize the following areas: (1) Enhancing key metabolic pathways: precisely reinforcing genes involved in the synthesis of organic acids such as lactate to optimize the fermentation process and accelerate the establishment of a dominant fermentation environment; (2) Broadening substrate utilization range: engineering strains to efficiently express fibrolytic enzymes (e.g., cellulases, xylanases), enhancing the degradation rate of lignocellulosic components in low-quality forage; (3) Inhibiting harmful contaminants: introducing or optimizing the biosynthesis of targeted antimicrobial compounds (e.g., bacteriocins, antifungal peptides) and key functional enzymes (e.g., mycotoxin-degrading enzymes) to precisely suppress spoilage microorganisms and reduce mycotoxin contamination. (4) Improving probiotic functionality: enhancing metabolic pathways in engineered strains for the synthesis of functional metabolites (e.g., GABA, flavonoids) to promote the accumulation of these beneficial components in silage. Through advances in these key areas, systematic optimization of the silage fermentation process is anticipated, ultimately achieving synergistic improvements in feed quality, resource utilization efficiency, and livestock health management. Looking ahead, this technology may facilitate the transition of animal husbandry towards healthy breeding and efficient production, providing innovative solutions for global food security and sustainable development goals.

### Technical challenges and development paths for the industrial application of CRISPR-engineered strains

Case studies in this review demonstrated the feasibility of employing CRISPR-Cas9 systems for precise genome editing in *Lactobacillus* to construct engineered strains with enhanced key traits. However, broader implementation of this technology requires development of more efficient and universally applicable gene-editing tools. Currently, the CRISPR systems still face challenges in certain lactic acid bacteria strains, including limited editing efficiency and delivery difficulties. There is need for vector design optimization, host range expansion, and editing precision improvement to facilitate industrial-scale applications of engineered strains.

Notably, the abundant native CRISPR-Cas systems in *Lactobacillus* species remain underutilized, representing valuable resources for developing strain-specific editing tools. Beyond technological breakthroughs, widespread adoption of CRISPR-engineered bacterial strains must address critical barriers in biosafety (genetic drift risks), regulatory frameworks (legal supervision), and social acceptance (GMO safety concerns). In summary, at the technical level, future studies should prioritize enhancing gene-editing efficiency, evaluating environmental release risks of engineered strains, and developing scaled-up production processes to accelerate practical implementation.

## Conclusions

The advancement of CRISPR-Cas9 genome editing technology presents a transformative opportunity for optimizing *Lactobacillus* strains to enhance silage quality and sustainability. By enabling precise genetic modifications, this approach overcomes the limitations of traditional strain screening methods, allowing for the development of tailored microbial inoculants with superior lactic acid production, enzymatic activity, and environmental adaptability. The integration of synthetic biology into silage fermentation not only improves feed preservation and nutrient retention but also enhances the probiotic functionality of the feed, thereby contributing to resource-efficient and healthy livestock production. Future research should focus on refining CRISPR-Cas9 toolkits for *Lactobacillus*, scaling up engineered strains for practical application, and assessing their long-term ecological and economic impacts. Embracing these innovations will be crucial for achieving sustainable agriculture and meeting the growing demands of animal food production.

## Data Availability

Not applicable.

## Abbreviations

CFU: Colony-forming units
CRISPR: Clustered Regularly Interspaced Short Palindromic Repeats
DBTL: Design-Build-Test-Learn
DMI: Dry matter intake
DON: Deoxynivalenol
DSB: Double strand break
dsDNA: Double-stranded DNA
EPS: Exopolysaccharides
GABA: γ-Aminobutyric acid
gRNA: Guide RNA
HDR: Homology-directed repair
HR: Homologous recombination
ILA: Indolelactic acid
LAB: Lactic acid bacteria
NHEJ: Non-homologous end joining
PAM: Protospacer adjacent motif
PLA: Phenyllactic acid
RE: Regulating element
SCFA: Short-chain fatty acid
ssDNA: Single-stranded oligonucleotides
TALENs: Transcription activator-like effector nucleases
WSC: Water-soluble carbohydrates
ZEN: Zearalenone
ZFNs: Zinc-finger nucleases
